# Decoherence Control of Nitrogen-Vacancy Centers

**DOI:** 10.1038/s41598-017-12280-z

**Published:** 2017-09-20

**Authors:** Chao Lei, Shijie Peng, Chenyong Ju, Man-Hong Yung, Jiangfeng Du

**Affiliations:** 1 Hefei National Laboratory for Physics Sciences at Microscale and Department of Modern Physics, University of Science and Technology of China, Hefei, 230026 China; 2 Synergetic Innovation Center of Quantum Information and Quantum Physics, University of Science and Technology of China, Hefei, Anhui 230026 People’s Republic of China; 3 Department of Physics, The University of Texas at Austin, Austin, Texas 78712 USA; 4 Institute for Quantum Science and Engineering and Department of Physics, Southern University of Science and Technology, Shenzhen, 518055 People’s Republic of China

## Abstract

Quantum mechanical systems lose coherence through interacting with external environments—a process known as decoherence. Although decoherence is detrimental for most of the tasks in quantum information processing, a substantial degree of decoherence is crucial for boosting the efficiency of quantum processes, for example, in quantum biology and other open systems. The key to the success in simulating those open quantum systems is therefore the ability of controlling decoherence, instead of eliminating it. Motivated by simulating quantum open systems with Nitrogen-Vacancy centers, which has become an increasingly important platform for quantum information processing tasks, we developed a new set of steering pulse sequences for controlling various coherence times of Nitrogen-Vacancy centers; our method is based on a hybrid approach that exploits ingredients in both digital and analog quantum simulations to dynamically couple or decouple the system with the physical environment. Our numerical simulations, based on experimentally-feasible parameters, indicate that decoherence of Nitrogen-Vacancy centers can be controlled externally to a very large extend.

## Introduction

A quantum simulator^1–5^ is potentially a powerful tool for solving many-body problems that are not tractable by classical methods. Generally, there are two types of quantum simulators. The first type, called digital quantum simulator^2–11^, makes use of a general-purpose quantum computer, where quantum states are encoded with qubits and the dynamical evolution is programmed in a quantum circuit. The other kind of quantum simulators are called analog quantum simulators^12–16^, where the Hamiltonian of the simulated quantum system is directly engineered in a dedicated quantum device, for example, trapped ions^14,17^ and optical lattices^18^.

The main challenge of constructing a practical quantum simulator is to reduce the influence of environmental decoherence, a universal problem for all tasks in quantum information processing including quantum communication^19^. In practice, a quantum simulator is necessarily an *open quantum system*, where the underlying system-environment interaction^20^ plays the main role in determining the performance of a quantum simulator. Furthermore, it is important to understand how a quantum simulator can simulate open quantum systems^20^, which is also intractable since the large number of degree of environment but of fundamental importance for understanding many physical phenomena in, for example, quantum optics^21^, quantum measurement^22^, and biological systems^23^.

In the literature, digital approaches of open-system quantum simulation^24–27^ have been theoretically studied and experimentally demonstrated. Similarly, analog quantum simulators of open quantum system has been theoretically proposed^28^ and experimentally investigated^29^. An important approach to tackle the decoherence problem, called dynamical decoupling^30–36^, has been developed to significantly eliminate the system-environment interactions^37^, through a sequence of external pulses applied to the system. Experimental implementations of dynamical decoupling indicate that such approach is widely applicable to various experimental platforms^38–45^. Moreover, an extension of dynamical decoupling is possible for universal quantum computation^46,47^, and other applications^48,49^.

As one of the most promising platform of quantum computation and quantum simulation^50–53^, Nitrogen-Vacancy(NV) center in diamond can be manipulated in room temperature with a long coherence time. NV centers have been proposed to simulate various quantum systems and realized in experiment, for example Helium Hydride Cation^51^ and directly measuring topological quantum number^53^. Here we study the possibility of simulating *open* quantum systems through an extension of the idea of dynamical decoupling and apply it to NV centers. Dynamical decoupling has been applied to NV center for eliminating the noise from environment and thus prolong the coherence time of electron spin^40,54,55^. In this paper, we developed a new set of decoupling pulse sequences that can control the decoherence of the off-diagonal matrix elements of the system density matrix.

The pulse sequences of interest in this work are different from those in dynamical decoupling^30–32^, whose primary goal is to *decouple* the system from the noise of the environment. In other words, the goal of dynamical decoupling was to maintain the purity of the quantum system. Here we aim to *control* the decoherence by exploiting the existing environment, so that we can simulate the dynamics of an open quantum system without the need to maintain the purity of the system, instead, environment of the quantum simulator can be employed to be as part of open quantum system. Consequently, we can avoid the need of including extra ancilla qubits as in other digital approaches of simulating open quantum systems.

## Results

### Full system controllability

A NV center can be viewed as a 3-dimensional qudit with a Hamiltonian *H*_*NV*_ of the following form^40,54,55^:

1 $${H}_{NV}=D{S}_{z}^{2}+\gamma {B}_{z}{S}_{z},$$

where *S*_*z*_ is the 3-dimensional spin operator for the spin-1 particle {*m* = 1,0,−1}, *D* is the zero-field splitting, *γ* = *gμ*_*B*_ is the gyromagnetic ratio with *μ*_*B*_ the Bohr magneton, *g* is the g-factor of electron, and *B*_*z*_ is the static magnetic field applied along *z* direction ([111] axis). Before we demonstrate how to control decoherence, we first show how to simulate a general 3-dimensional system with a NV center in rotating frame.

For any given Hamiltonian $\hat{H}$ and quantum state $|{\rm{\Psi }}\rangle$, and a unitary operator $\hat{U}={e}^{-i\hat{A}t}$ associated with a self-adjoint operator $\hat{A}$ (where we used the natural units that set $\hslash \mathrm{=1}$), the corresponding quantum state in the rotating frame is given by, $|{{\rm{\Psi }}}_{{\rm{rot}}}\rangle \equiv {\hat{U}}^{\dagger }|{\rm{\Psi }}\rangle$. We can obtain an effective Hamiltonian *H*_*rot*_ in the rotating frame as follows: ${H}_{{\rm{rot}}}={\hat{U}}^{\dagger }\hat{H}\hat{U}-\hat{A}$. In our case, we include two sets of microwave pulses to a NV center. In the laboratory frame, the Hamiltonian in Eq. ((1)) becomes:

2 $${H}_{NV}^{mw}={H}_{NV}+\gamma {B}_{1}{S}_{x}cos\,{\omega }_{1}t+\gamma {B}_{2}{S}_{x}cos\,{\omega }_{2}t,$$

where *H*_*NV*_ is the Hamiltonian shown as in Eq. ((1)), *S*_*x*_ is a 3 × 3 spin operator, *B*_1/2_ is the magnetic field amplitude of the applied microwave along the $\hat{x}$ axis, and *ω*_1_ and *ω*_2_ are the frequencies of the applied microwave. The target Hamiltonian *H*_*S*_ can be obtained by choosing a rotating frame reference where $\hat{A}={\omega }_{1}|1\rangle \langle 1|+{\omega }_{2}|-1\rangle \langle -1|$, which gives the following simulated Hamiltonian:

3 $${H}_{S}=[\begin{array}{ccc}D+\gamma {B}_{z}-{\omega }_{1} & \gamma {B}_{1}\mathrm{/2}\sqrt{2} & 0\\ \gamma {B}_{1}\mathrm{/2}\sqrt{2} & 0 & \gamma {B}_{2}\mathrm{/2}\sqrt{2}\\ 0 & \gamma {B}_{2}\mathrm{/2}\sqrt{2} & D-\gamma {B}_{z}-{\omega }_{2}\end{array}]$$

If the microwaves are turned off, then *B*_1_ = *B*_2_ = 0, while in the resonance conditions: *ω*_1_ = *D* + *γB*_*z*_ and *ω*_2_ = *D* − *γB*_*z*_, the system Hamiltonian is *H*_*S*_ = 0.

### Noises in Nitrogen-Vacancy (N-V) centers

In the process of diamond growth, impurities such as isotope ^13^C and nitrogen sneak inside. The most possible result of substituting Nitrogen atoms is to generate P1 centers(while NV centers is a P1 centers that captured a vacancy). Normally, a NV center is subject to a local environment dominated by the surrounding nuclear spins of ^13^C’s and electron spins of P1 centers, which effectively produce a random magnetic field *b*(*t*) to the NV center^54,40,56^, i.e.,

4 $${H}_{SB}=\gamma b(t){S}_{z}=\gamma b(t)(|1\rangle \langle 1|-|-1\rangle \langle -1|)$$

which is also the system-environment interaction Hamiltonain of the simulated quantum system, since in rotating frame *H*_*SB*_ remains the same as in laboratory frame.

For the case where the environment is dominated by the nuclear spins, the random fluctuation of the magnetic field can be regarded as stationary, i.e., *b*(*t*) = *b*, and is usually approximated as Markovian and Gaussian^54^, i.e., with a probability distribution $Pr(b)={e}^{-{b}^{2}\mathrm{/2}{\sigma }_{b}^{2}}/\sqrt{2\pi }{\sigma }_{b}$, where *σ*_*b*_ is the variance of the random magnetic field from the spin bath. For the case where the noise come from electron spins instead, the random process of *b*(*t*) can be approximated by the Ornstein-Uhlenbeck process^40^, with a correlation function *C*(*t*) given by the follow: $C(t)=\langle b\mathrm{(0)}b(t)\rangle ={l}^{2}\exp \,(-R|t|)$, where *l* describes the characteristic strength of the coupling of the NV center to the bath, and *R* = 1/*τ*_*c*_ is the transition rate, with *τ*_*c*_ being the correlation time of the spin bath^40^.

### Strengthening decoherence

We note that the evolution operator *e* ^−*iHt*^ of the total system can be divided by many small time slices, Δ*t* ≡ *t*/*n*, ${e}^{-iHt}={lim}_{n\to \infty }{({e}^{-i{H}_{S}{\rm{\Delta }}t}{e}^{-i{H}_{SB}{\rm{\Delta }}t}{e}^{-i{H}_{B}{\rm{\Delta }}t})}^{n}$ in which *H*_*B*_ is the Hamiltonian of the environment which can be treated as interacting spin system^56^. One way to strengthen decoherence, assisted by the environment, can be achieved as follows: first, turn off the system Hamiltonian momentarily, for a time period, *λ*Δ*t*, where *λ* > 0, the evolution of the total system is then: ${e}^{-i({H}_{SB}+{H}_{B})\lambda {\rm{\Delta }}t}$, which can be realized by seting B_1/2_ = 0 and $\omega =D\pm \gamma {B}_{z}-\frac{\varepsilon }{1+\lambda }$ in Eq. (3), where *ω* is the frequency of the microwave and *ε* is the diagonal element of simulated quantum system, here the factor $\frac{1}{1+\lambda }$ appears as the diagonal elements do not disappear in the same rotating frame when turning off the microwaves. Then we allow the total system to evolve freely for a time period of Δ*t*. The pattern is then repeated for *n* times, i.e., ${({e}^{-i({H}_{S}+{H}_{SB}+{H}_{B}){\rm{\Delta }}t}{e}^{-i({H}_{SB}+{H}_{B})\lambda {\rm{\Delta }}t})}^{n}$, which can be appoximately rewritten as ${({e}^{-i({H}_{S}+\mathrm{(1}+\lambda )({H}_{SB}+{H}_{B})){\rm{\Delta }}t})}^{n}$ if Δ*t* is small enough.

Therefore, in the large-*n* limit, we obtain an effective Hamiltonian as follow:

5 $${H}_{{\rm{eff}}}={H}_{S}+\mathrm{(1}+\lambda )({H}_{SB}+{H}_{B}),$$

which contains an interaction term *H*_*SB*_ amplified by a factor of (1 + *λ*). The side product is that the environment Hamiltonian is also amplified, that there also appear a factor *λ* before the environment Hamiltonian *H*_*B*_. However, for the spin environments of NV centers, the effect can be ignored.

### Controlling noises from nuclear and electron spin baths

In the following we consider combining Trotter expansion with decoupling pulses to control the decoherence in NV centers. For NV centers in ultra-pure diamonds(in which there is no measurable nitrogen impurity), the dominant decoherence source comes from the surrounding ^13^C spin bath^54^, which is random but stationary within the time-scale of system dynamics. Consequently, for a qubit initialized in a pure state, $|{\psi }_{0}\rangle =\alpha |0\rangle +\beta |1\rangle$, and *H*_*S*_ = 0, the off-diagonal matrix element (or coherence), ${\rho }_{12}=\alpha {\beta }^{\ast }\int Pr(b){e}^{-i2b\mathrm{(1}+\lambda )t}db$, decays as follows:

6 $${\rho }_{12}=\alpha {\beta }^{\ast }{e}^{-2{\sigma }_{b}^{2}{\mathrm{(1}+\lambda )}^{2}{t}^{2}}$$

which implies that the effective coherence time *T*_2_ can be controlled by the parameter *λ*,

7 $${T}_{2}(\lambda )=\frac{1}{\sqrt{2}{\sigma }_{b}\mathrm{(1}+\lambda )}\mathrm{.}$$

**Figure 1:**
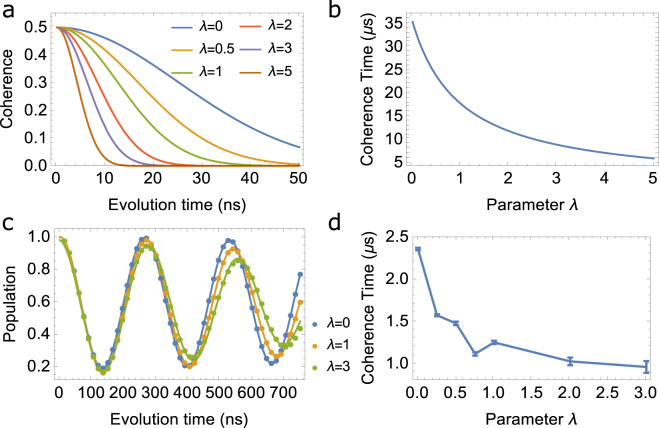
(**a**) Coherence vs evolution time with only nuclear spin noise, the variance of the random magnetic field is set to be $0.2$ Gauss. (**b**) Coherence time vs *λ* extracted from (**a**). (**c**)Evolution of population with time in real NV center, in which both nuclear spin noise and electron noise are considered, the dots are simulated points and the curves are fitted one. (**d**) Coherence time got from the fitting curve in (**c**) vs *λ* from 0 to 3, here the values of *λ* in the calculation are 0, 0.25, 0.5, 0.75, 1, 2, 3.

The results are shown in Fig. 1, from Fig. 1(a), it is clear that tuning the parameter *λ* can strengthen the decoherence, i.e., we can destroy the coherence via increasing the value of *λ*. The coherence time can be extract from above and shown in Fig. 1(b).

In diamonds with nitrogen impurities, such as Type I, the electron spins also contribute to the decoherence of the system, which means that both nuclear and electron noise should be included. We investigate the decoherence of a two-level system numerically with experimental parameters of NV center, in which both nuclear and electron spin noise are included. The simulated results are shown in Fig. 1(c) and (d), in which only one microwave is applied. The parameters are as follow: The static magnetic field is *B*_*z*_ = 100 Gauss, the zero-field splitting is *D* = 2.87*GHz*, the frequency of applied microwave is a little away from the resonance frequency(which is 3.15 GHz) of NV center at the given static magnetic field with a detuning 1.9 × 10^6^/(1 + *λ*) Hz and with a amplitude of 1.717 Gauss, *λ* is varied from zero (i.e. the ${T}_{2}^{\ast }$) to 3. the coherence time vs *λ* is shown in Fig. 1(d), it shows that the coherence time of NV center decrease when *λ* increases, which shows a effective control of decoherence caused by system-environment interaction.

### Weakening decoherence

**Figure 2:**
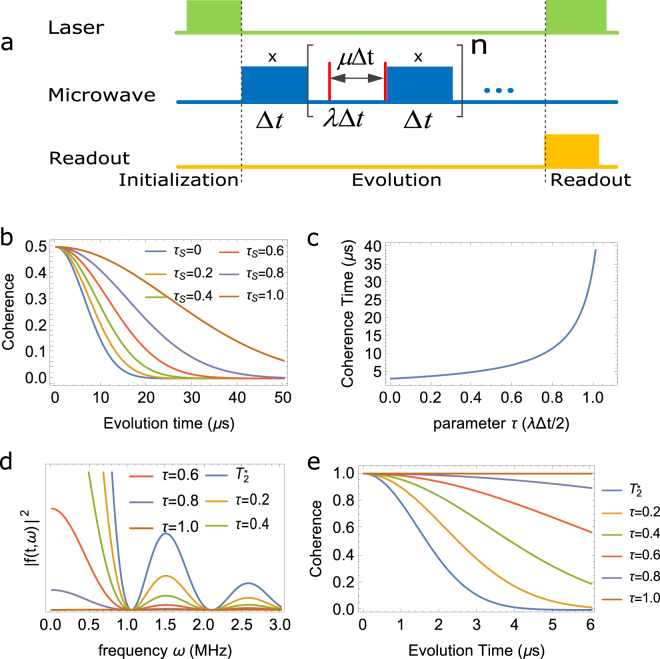
(**a**) Pulse sequence for two-level system, where $x$ means the microwave is applied along $x$ axis, the red bar is the idea $\pi$ pulse (i.e. the swap gate), blue rectangles mean the evolution of ${H}_{S}$, which in the numerical calculations we always set it to be 0 without losing generalization. (**b**) Coherence vs evolution time with different distance between two swap gate, the variance of the random magnetic field is set to be 0.2 $Gauss$. (**c**) Coherence time vs *τ*, which is defined in $\mu {\rm{\Delta }}t=\tau \lambda {\rm{\Delta }}t\mathrm{/2}$. (**d**) is the values of $|\tilde{f}(t,\omega {)|}^{2}$ which represent the noise spectrum. (**e**) The coherence vs time with different *τ*.

Let us consider a two-level system and a swap gate defined by: $${u}_{12}\equiv {\sigma }_{x}=(\begin{array}{cc}0 & 1\\ 1 & 0\end{array})$$, after which the two state applied undergo an opposite evolution under the influence of environment noise and thus decouple the quantum system from the environment(i.e. the microwave is turned off) after two swap gates are inserted, shown as in Fig. 2(a). During the decoupling part of Trotter decomposition,the evolution is then:

8 $${\sigma }_{x}{e}^{-i{H}_{SB}{t}_{2}}{\sigma }_{x}{e}^{-i{H}_{SB}{t}_{1}}={e}^{-i{H}_{SB}({t}_{1}-{t}_{2})}$$

where we set *t*_1_ = (*λ* − *μ*)Δ*t* and *t*_2_ = *μ*Δ*t*. The overall evolution becomes ${({e}^{-i({H}_{S}+{H}_{SB}){\rm{\Delta }}t}{e}^{-i{H}_{SB}(\lambda -2\mu ){\rm{\Delta }}t})}^{n}$, which implies the following effective Hamiltonian:

9 $${H}_{{\rm{eff}}}={H}_{S}+\mathrm{(1}+\lambda -2\mu ){H}_{SB}$$

Supposing a qubit initialized in a pure state, $|{\psi }_{0}\rangle =\alpha |0\rangle +\beta |1\rangle$, and *H*_*S*_ = 0 and the environment noise is quasi-static, for the case in NV center, it corresponds to the case that the noise come from the nuclear spin, the coherence vs evolution time under Eq. ((9)) becomes:

10 $${\rho }_{12}=\alpha {\beta }^{\ast }{e}^{-2{\sigma }_{b}^{2}{\mathrm{(1}+\lambda -2\mu )}^{2}{t}^{2}}$$

This is shown in Fig. 2(b) and the coherence time is:

11 $${T}_{2}^{dd}=\frac{1}{\sqrt{2}{\sigma }_{b}\mathrm{(1}+\lambda -2\mu )},$$

which is shown in Fig. 2(c). Here we set the distance between two swap gate as *μ*Δ*t* = *τλ*Δ*t*/2, where *τ* is the variable shown in Fig. 2 that $\tau =\frac{\mu }{\lambda \mathrm{/2}}$. When *τ* = 0, it is equal to the case with no decoupling pulse, but when *τ* = 1, the distance between the two swap gate is half of the *λ*Δ*t*, which is the same as the CPMG pulse^57,58^.

In the presence of electron spin noise, the coherence factor of a two-level system subject to dynamical decoupling is given^59^ by, $W(t)=|\langle \exp (-i{\int }_{0}^{t}b(t^{\prime} )f(t;t^{\prime} )dt^{\prime} )\rangle |={e}^{\chi (t)}$, where *b*(*t*) is the random noise, the function *f*(*t*;*t*′) depends on the pulse sequence as:

12 $$f(t;t^{\prime} )=\sum _{k\mathrm{=0}}^{n}{(-\mathrm{1)}}^{k}\theta ({t}_{k+1}-t^{\prime} )\theta (t^{\prime} -{t}_{k})\,,$$

with *θ*(*t*′) the Heaviside step function, *t*_0_ = 0 and ${t}_{n+1}=t$ is the total evolution time.

Furthermore, the spectral density of the noise *C*(*ω*) is given^40^ by, $C(\omega )={l}^{2}\frac{2R}{{R}^{2}+{\omega }^{2}}$, which implies that

13 $$\chi (t)={\int }_{0}^{\infty }\frac{d\omega }{2\pi }C(\omega ){|\tilde{f}(t,\omega )|}^{2}\,,$$

where $\tilde{f}(t,\omega )={\int }_{-\infty }^{\infty }{e}^{i\omega t}f(t;t^{\prime} )dt$. For our case, the square of Fourier transform of *f*(*t*; *t*′) is found to be:

14 $$|\tilde{f}(t,\omega {)|}^{2}=\frac{1}{{\omega }^{2}}\frac{1-\,\cos \,\omega t}{1-\,\cos \,\omega \delta }\mathrm{(6}+2\,\cos \,\omega \delta -4\,\cos \,\omega {\delta }_{1}-4\,\cos \,\omega {\delta }_{2})$$

where *δ* = *λ*Δ*t*, *δ*_1_ = (*λ* − *μ*)Δ*t* and *δ*_2_ = *μ*Δ*t*, Eq. (14) is the spectral dependence of decoupling pulses and is known as “Filter function”. The values of $|\tilde{f}(t,\omega {)|}^{2}$ are shown in Fig. 2(d) with different values of *τ*, which is defined in the following relation: *μ*Δ*t* = *τλ*Δ*t*/2. We obtained the coherence factor shown as in Fig. 2(e), after applying a large cut-off frequency. It is obviously that when *τ* becomes larger, the coherence time becomes longer.

### Fine-tuning decoherence for qudit

**Figure 3:**
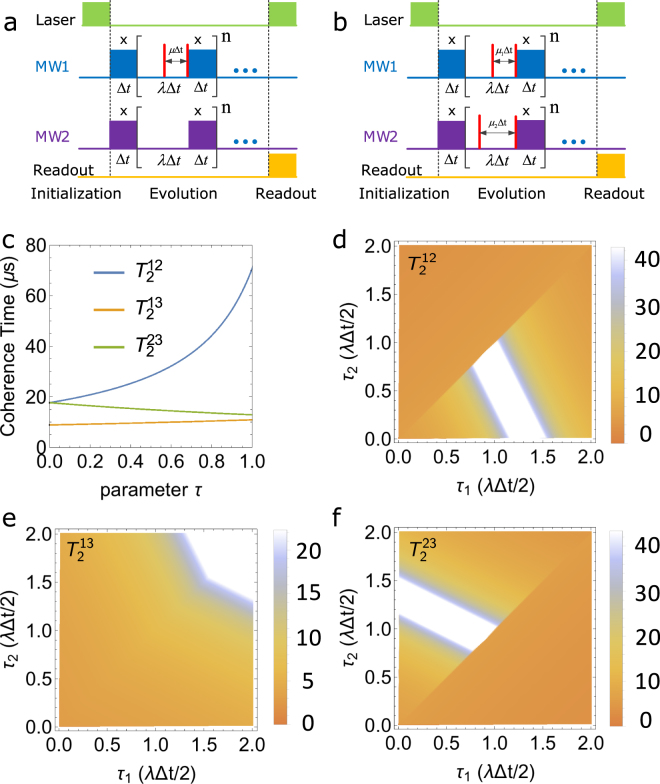
(**a**) Pulse sequences for the case applying the decoupling pulses in only one channel in three-level system, labels have the same meaning as Fig. 1. (**b**) Pulse sequences of the case that applying the decoupling pulses in two channel of the three-level system, where $MW1$ and $MW2$ mean the first and second microwave with different frequencies. (**c**)The coherence time vs *τ*(which is defined in $\mu {\rm{\Delta }}t=\tau \lambda {\rm{\Delta }}t\mathrm{/2}$), as *τ* increases, the coherence time ${T}_{2}^{12}$ increases and the other two coherence time remains almost the same, decrease or increase slightly. (**d**–**f**) show the coherence time between the three levels, with (**d**) is the ${T}_{2}^{12}$, (**e**) the ${T}_{2}^{13}$ and (**f**)${T}_{2}^{23}$, in which *τ*_1_ and *τ*_2_ are defined in ${\mu }_{\mathrm{1(2)}}{\rm{\Delta }}t={\tau }_{\mathrm{1(2)}}\lambda {\rm{\Delta }}t\mathrm{/2}$, the blue region means the coherence time is small and the red ones is the case the coherence time increases sharply.

For general multi-level systems, i.e., qudit, we have an extra tool to fine-tuning the decoherence for different off-diagonal elements in the density matrix. Here we consider only the stationary noise from nuclear spin in three-level system. Let us consider that the dynamical decoupling pulses, ${u}_{12}=|1\rangle \langle 0|+|0\rangle \langle 1|+|-1\rangle \langle -1|$, are applied on only one channel(shown as in Fig. 3(a)), between $|m=1\rangle$ and $|m=0\rangle$, then the relevant part in evolution operator becomes:

15 $${u}_{12}{e}^{-i{H}_{SB}{t}_{2}}{u}_{12}{e}^{-i{H}_{SB}{t}_{1}}\equiv {e}^{-ib(t)L({t}_{1},{t}_{2})},$$

where $L({t}_{1},{t}_{2})=|1\rangle \langle 1|{t}_{1}+|0\rangle \langle 0|{t}_{2}-|-1\rangle \langle -1|({t}_{1}+{t}_{2})$, we again set: ${t}_{1}=(\lambda -\mu ){\rm{\Delta }}t$ and ${t}_{2}=\mu {\rm{\Delta }}t$, which gives ${t}_{1}+{t}_{2}=\lambda {\rm{\Delta }}t$ shown as in Fig. 3(a).

Therefore, when we choose ${t}_{1} < {t}_{2}$, we effectively make state $|1\rangle$ experience less dephasing then state $|0\rangle$, and vice versa. For any given initial state, ${\psi }_{0}=\alpha |1\rangle +\beta |0\rangle +\gamma |-1\rangle$, if we set ${H}_{S}=0$, then the off diagonal elements of the associated density matrix decay as follows:

16 $${\rho }_{12}=\alpha {\beta }^{\ast }{e}^{-{\sigma }_{b}^{2}{\mathrm{(1}+\lambda -2\mu )}^{2}{t}^{2}\mathrm{/2}}$$

17 $${\rho }_{13}=\alpha {\gamma }^{\ast }{e}^{-{\sigma }_{b}^{2}{\mathrm{(2}+2\lambda -\mu )}^{2}{t}^{2}\mathrm{/2}}$$

18 $${\rho }_{23}=\beta {\gamma }^{\ast }{e}^{-{\sigma }_{b}^{2}{\mathrm{(1}+\lambda +\mu )}^{2}{t}^{2}\mathrm{/2}}$$

here the lower label 1, 2, 3 represent the state $|m=1\rangle$, $|m=0\rangle$ and $|m=-1\rangle$, also this can be got after averaging of environment noise which is stationary Gaussian. In other words, the coherence times of the off-diagonal elements are given by:

19 $$\begin{array}{l}{T}_{2}^{12}=\frac{\sqrt{2}}{{\sigma }_{b}\mathrm{(1}+\lambda -2\mu )}\\ {T}_{2}^{13}=\frac{\sqrt{2}}{{\sigma }_{b}\mathrm{(2}+2\lambda -\mu )}\\ {T}_{2}^{23}=\frac{\sqrt{2}}{{\sigma }_{b}\mathrm{(1}+\lambda +\mu )}\end{array}$$

The dependence of the coherence times with the parameter *μ* is shown in Fig. 3(c). We see that the coherence time ${T}_{2}^{12}$ is more sensitive to the change of *μ*, compared with the other coherence times.

Furthermore, additional dynamical decoupling pulses, e.g., ${u}_{23}=|1\rangle \langle 1|+|-1\rangle \langle 0|+|0\rangle \langle -1|$, can be applied, ${u}_{12}$ and ${u}_{23}$ are the $\pi$ pulse applied between $|m=-1\rangle \leftrightarrow |m=0\rangle$ and $|m\mathrm{=0}\rangle \leftrightarrow |m\mathrm{=1}\rangle$ respectively, then we have the dynamics of the system as(let ${\mu }_{2} > {\mu }_{1}$):

20 $${u}_{23}\,{u}_{12}\,{e}^{-i{H}_{SB}{t}_{3}}{u}_{12}{e}^{-i{H}_{SB}{t}_{2}}{u}_{23}{e}^{-i{H}_{SB}{t}_{1}}\equiv {e}^{-ib(t)L({t}_{1},{t}_{2},{t}_{3})}$$

where $L({t}_{1},{t}_{2},{t}_{3})=|1\rangle \langle 1|({t}_{1}+{t}_{2})-|0\rangle \langle 0|({t}_{2}+{t}_{3})-|-1\rangle \langle -1|({t}_{1}-{t}_{3})$. Suppose we set ${t}_{1}=(\lambda -{\mu }_{2}){\rm{\Delta }}t$, ${t}_{2}=({\mu }_{2}-{\mu }_{1}){\rm{\Delta }}t$ and ${t}_{3}={\mu }_{1}{\rm{\Delta }}t$ (shown in Fig. 3(b) with $0\le {\mu }_{1}\le \lambda$ and ${\mu }_{1}\le {\mu }_{2}\le \lambda$. It gives ${t}_{1}+{t}_{2}+{t}_{3}=\lambda {\rm{\Delta }}t$. Consequently, for any initial state, ${\psi }_{0}=\alpha |1\rangle +\beta |0\rangle +\gamma |-1\rangle$, the time-dependent off-diagonal elements of the density matrix are given by:

21 $$\begin{array}{rcl}{\rho }_{12}(t) & = & \alpha {\beta }^{\ast }{e}^{-\frac{1}{2}{\sigma }_{b}^{2}{\mathrm{(1}+\lambda -({\mu }_{1}-{\mu }_{2}))}^{2}{t}^{2}}\\ {\rho }_{13}(t) & = & \alpha {\gamma }^{\ast }{e}^{-\frac{1}{2}{\sigma }_{b}^{2}{\mathrm{(2}+2\lambda -\mathrm{(2}{\mu }_{1}+{\mu }_{2}))}^{2}{t}^{2}}\\ {\rho }_{23}(t) & = & \beta {\gamma }^{\ast }{e}^{-\frac{1}{2}{\sigma }_{b}^{2}{\mathrm{(1}+\lambda -({\mu }_{1}+2{\mu }_{2}))}^{2}{t}^{2}}\end{array}$$

which means that the coherence times of the off-diagonal elements are given by:

22 $$\begin{array}{rcl}{T}_{2}^{12} & = & \frac{\sqrt{2}}{{\sigma }_{b}\mathrm{(1}+\lambda -({\mu }_{1}-{\mu }_{2}))}\\ {T}_{2}^{13} & = & \frac{\sqrt{2}}{{\sigma }_{b}\mathrm{(2}+2\lambda -\mathrm{(2}{\mu }_{1}+{\mu }_{2}))}\\ {T}_{2}^{23} & = & \frac{\sqrt{2}}{{\sigma }_{b}\mathrm{(1}+\lambda -({\mu }_{1}+2{\mu }_{2}))}\end{array}$$

The coherence times with parameters ${\mu }_{\mathrm{1(2)}}$ are shown in Fig. 3(d–f)(in which the case of ${\tau }_{2}\le {\tau }_{1}\le 2$ is also included, where *τ*_1_ and *τ*_2_ are defined in ${\mu }_{\mathrm{1(2)}}{\rm{\Delta }}t={\tau }_{\mathrm{1(2)}}\lambda {\rm{\Delta }}t\mathrm{/2}$). In the plots, the orange region means the coherence time is small and the blue-white one is the case the coherence time increases sharply. It shows that the coherence between different levels can be tuned though changing the insert time of the two decoupling time, which is similar with the case of only one channel is applied with decoupling pulse. For the case that decoupling pulses are applied on both channels, the coherence time ${T}_{2}^{13}$ increases when ${\mu }_{1}$ and ${\mu }_{2}$ are close to *λ* (shown in Fig. 3(e)), while the other two coherence time remains essentially the same as the one before applying the decoupling pulses, and here the coherence time of other two state without decoupling pulses applied increase at a range that is different from the other channels, this shows that with decoupling pulses applied only two channels, we can get the coherence time between all the states tuned.

## Discussions

Trotter decomposition combined with decoupling pulses is shown to be able to control the decoherence in NV centers, although this method is numerically tested only for NV centers with two and three energy levels, further generalization of our method to $d$-dimensional ($d\ge 4$) systems is possible. Following the previous results, the decoherence of different off-diagonal elements can be controlled by the following sequence:

23 $$\prod _{i < j}{u}_{ij}\prod _{i < j}[{e}^{-i{H}_{SB}{t}_{ij}}{u}_{ij}]{e}^{-i{H}_{SB}{t}_{0}}{e}^{-i({H}_{S}+{H}_{SB}){\rm{\Delta }}t},$$

where ${t}_{ij}$’s are the adjustable waiting time before the swap gate between $i$ and $j$ level is applied after the next swap gate ${u}_{ij}=I+|i\rangle \langle j|+|j\rangle \langle i|-|i\rangle \langle i|-|j\rangle \langle j|$, where ${t}_{0}$ is included as the waiting time before the first swap gate applied.

In conclusions, in this work, we have presented a new method that can control the decoherence in NV center by combining the Trotter decomposition and decoupling pulses, which have potential applications in simulating open quantum system with NV center. The scheme exploits the intrinsic decoherence from the environment, and contains the benefits of the university of digital quantum simulation and also the efficiency of analog quantum simulation. Our results indicate that such a scheme is experimentally feasible. This method could also be used as a test bed for non-Markovian open quantum systems^60,61^, without obvious roadblock. And also the method we proposed here can be applied to quantum simulation of various open quantum system, if it has the same type of environment of the quantum simulator, a simplest classification is Markovian or non-Markovian for example.

## Methods

### Model of environment in Nitrogen-Vancancy center

The environment we take into consideration contains both electron spins (mainly come from P1 center, i.e. the neutral charge state of substitutional Nitrogen) and nuclear spins (^13^C), but mainly nuclear spins, which is more similar to the exact bath of deep (about 10 *μm*) NV centers in diamond with a nitrogen content whose concentration is small than 1 ppb. The model used in this paper come from the literatures^40,54^, for the noise of nuclear spin, it was treated to be stationary compared with the evolution of the NV center, while for the electron spin noise, it is decribed as the Ornstein-Uhlenbeck process.

### Numerical calculations

The algorithm adopted here for Fig. 1(c) is RK4 (Runge-Kutta method of the 4-th order), which is a well-known numerical method of ordinary differential equations. In the calculations we used ideal $\pi$ pulse (i.e. the swap gates), while in laboratory frame, finite-time length of microwave is used to inverse the population of the corresponding two level if needed.

### Simulating a open quantum system in NV center

The simulation of the quantum system is realized in a rotating frame. The process is as follow: 1, Initialize the NV center to the $\mathrm{|0}\rangle$ state, from which the population can be read with laser, all of the other states can be read by transfering the population to this state; 2, Apply the microwave which is used to realize the simulating of the target quantum system, evolution for a time duration $\delta t$, during which both the quantum system and the environment are included; 3, Turn off the microwave for a duration of *λδt*, then only the environment is included in the evolution; 4, Repeat this process for n times, and finally with Trotter theorem, the strengthening of the system-environment interaction can be realized. To weaken the decoherence of the simulated quantum system, two $\pi$ pulses are applied to decouple the system from the environment, and the two pulses are inserted during the same period of evolution when the simulated quantum system is turned off.
